# Invasive Fungal Infections of the Head and Neck: A Tertiary Hospital Experience

**DOI:** 10.3390/pathogens13070530

**Published:** 2024-06-23

**Authors:** Tieying Hou, W. Robert Bell, Hector Mesa

**Affiliations:** 1 Department of Laboratory Medicine and Pathology, Division of Head & Neck Pathology, Indiana University School of Medicine, Indianapolis, IN 46202, USA; thou@iu.edu; 2 Department of Laboratory Medicine and Pathology, Division of Neuropathology, Indiana University School of Medicine, Indianapolis, IN 46202, USA; rb27@iu.edu

**Keywords:** invasive fungal infection, head and neck infections, risk factors, prognostic factors, treatment outcome

## Abstract

From the existing millions of fungal species, only a few cause disease. In this study, we investigated invasive fungal infections in the head and neck (H&N) over a 19-year period (2005 to 2024) at a large academic healthcare system. Among the 413 documented fungal H&N infections, 336 were noninvasive, and 77 were invasive. The highest incidence of invasive infections occurred in the sinonasal cavities, with a 15-fold difference compared to other sites. Most infections affected adults over 40 years old. The most common organisms were Mucorales (51%), hyaline molds (29%), and *Candida* (11%). Risk factors included malignancy, transplant, diabetes, and illicit drug use. Mortality was high in patients with malignancy and/or transplant. Infections affecting the mandible were usually a complication of osteoradionecrosis and were associated with the coinfection of Candida and Actinomyces. At other sites, infections were rare and were usually the result of penetrating injuries or immunosuppression. Treatment typically involved a combination of antifungals and surgical procedures.

## 1. Introduction

The number of fungal species is estimated at 2.2 to 3.8 million worldwide; however, of these, only about 120,000 have been scientifically classified [1], and only 19 are considered “priority pathogens” by the World Health Organization [2]. Superficial fungal infections affecting the skin and mucosae are bothersome but not life-threatening. By contrast, invasive infections are associated with necrosis or tumor-forming granulomatous reactions that can lead to significant tissue damage or a mass effect and be potentially life-threatening, depending on their extent and/or location, especially in individuals with weakened immune systems. Of the several million fungal species, the few genera most commonly causing invasive infections are *Candida, Aspergillus, Rhizopus, Mucor, Cryptococcus, Pneumocystis, Histoplasma, Coccidioides and Blastomyces*. The incidence of invasive fungal disease has been rising; recent assessments show that globally, every year, more than 150 million people suffer from serious infections and more than 1.6 million die of invasive mycoses [3]. The rise in the burden of invasive fungal disease has been attributed to the following: (1) the persistence of large populations affected by HIV/AIDS in Africa, (2) the expansion of immunosuppressive therapies worldwide, (3) the global emergence of multi-drugs resistant fungi, (4) the lack of rapid and sensitive diagnostics for the early recognition and accurate diagnosis of aggressive fungal infections, (5) the restricted availability of antifungal susceptibility testing and (6) the limited availability of antifungal agents. Despite modest recent progress in diagnosing and treating invasive fungal disease, the mortality rate remains high [4].

Invasive fungal infections of the head and neck (H&N) comprise a heterogenous group of diseases with diverse clinical presentations, treatment, and prognosis. The available literature mostly describes the involvement of the sinonasal passages in immunocompromised patients, which is associated with a high mortality rate of ~50% [5]. Timely diagnosis followed by surgical intervention, systemic antifungal therapy and the restoration of immune function, when possible, are crucial for improving clinical outcomes [6]. The literature involving other H&N sites, such as the oral cavity, pharynx, larynx, orbit or base of skull, is sparse and limited to case reports. In this study, we reviewed 413 cases of fungal infection at different H&N sites from a large tertiary university hospital system. The clinical presentation, imaging studies and pathologic findings were summarized and analyzed.

## 2. Materials and Methods

After IRB approval (IRB protocol # 10391), a natural language search of the pathology database of the Indiana University School of Medicine for the terms nasal/paranasal sinus, ear/mastoid, oral cavity, oropharynx/esophagus, base of skull and orbit fungal infections for the period 2005–2024 was performed. Inclusion criteria included a specific diagnosis of fungal infection (e.g., candidiasis, mycetoma, histoplasmosis) identified on hematoxylin eosin or special stains. Only cases explicitly described as invasive were included in the invasive category. Cases described as “contaminant, commensal, saprophytic, incidental” were excluded. Data extracted from the electronic medical records included demographic information, risk factors, the extent of the disease, treatments received, and outcome. Descriptive statistics on the extracted data were performed using IBM SPSS Statistics for Windows, Version 28.0. (2021 Armonk, NY USA: IBM Corp.).

## 3. Results

413 cases of fungal infections at different H&N sites were found, including 336 non-invasive and 77 invasive cases (Table 1). The ratio of non-invasive to invasive cases varied markedly by site, being the highest at the oropharynx/esophagus and lowest at the ear/mastoid (Table 1). The most common site of invasive fungal infections was the sinonasal cavities by a ratio of ≥15:1 compared to the other sites.

The cohort with invasive sinonasal infections consisted of 45 cases, 31 males and 14 females with an M:F ratio of 2.2. The mean age was 51.2 yrs. Only 3/45 (7%) of the cases affected children/adolescents <18 yrs., 9/45 (20%) affected young patients between 18 and 40 yrs., and the majority 33/45 (73%) affected adults >40 yrs. (Table 2).

**Figure 1:**
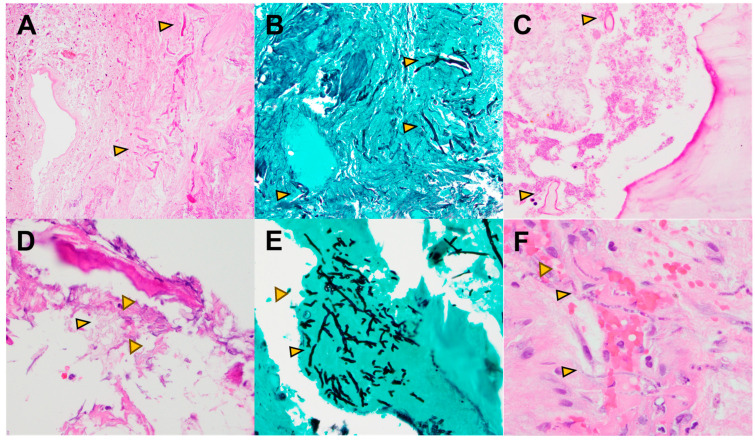
(**A**–**C**): Sinonasal mucormycosis. H&E (**A**) and GMS stain (**B**) show numerous fungal hyphae (arrowheads) invading necrotic sinonasal mucosa, including vascular walls (angioinvasion). H&E (**C**) shows nonseptate thick fungal hyphae (arrowhead) with irregular branching points invading necrotic sinonasal bone. (**D**–**F**): Aspergillosis. H&E (**D**) and GMS stain (**E**) reveal septate hyphae with acute angle or dichotomous branching, invading bone. Fungi are inconspicuous on H&E but become evident with GMS staining. H&E (**F**) shows fungal hyphae (arrowheads) invading vascular spaces in the submucosa.

Although cultures were collected in all the cases, the final identification of the fungal organism was carried out by morphology alone in 18/45 (40%) due to no growth and by culture in 27/45 (60%). A single organism was identified in 40/45 (89%) and >1 fungus in 5/45 (11%). The most common fungi were from the order Mucorales (28/45 (51%)), of which *Mucor* sp. was the most common (18/45 (64%)), followed by hyaline molds (16/45 (29%)), of which *Aspergillus* sp. was the most common (14/45 (88%)) (Figure 1), and *Candida*: 6/45 (11%) (Table 3).

**Figure 2:**
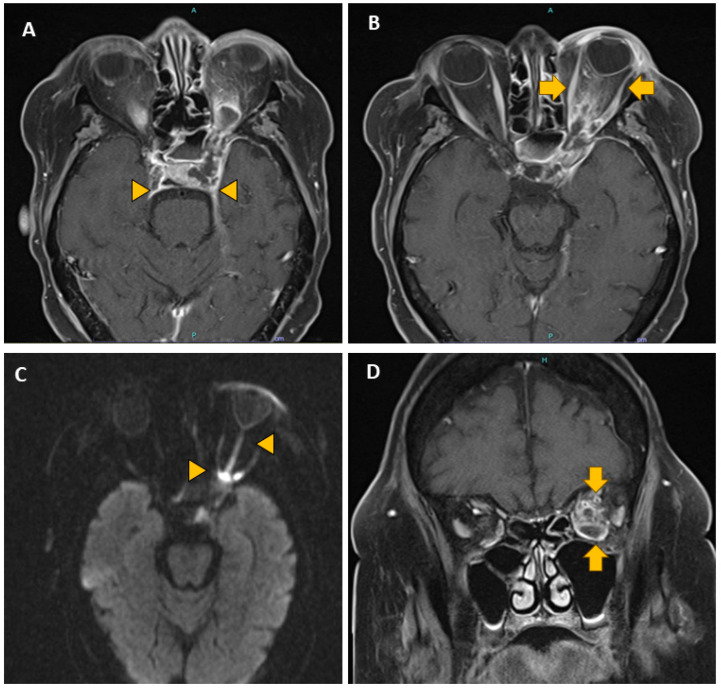
Magnetic resonance imaging, invasive fungal sinusitis with orbital and base of skull involvement. (**A**). Axial image showing left sphenoid sinus mucosal thickening with internal fluid and erosion of the base of skull (arrowheads). (**B**). Axial image showing left-sided proptosis and edema in the soft tissues (arrows), extending from the ocular globe to the orbital apex and cavernous sinus. (**C**). Axial T2-weighted image showing edema of the intraorbital optic nerve (arrow heads). (**D**). Coronal T2-weighted image showing extensive edema in the posterior orbit (arrows) involving soft tissues, the optic canal, vessels, and oculomotor nerves (orbital apex syndrome).

On clinical and radiological evaluation, only 12/45 (27%) patients had invasive infections limited to the sinonasal cavities, while the majority, 33/45 (73%), had infections extending into the surrounding tissues, most commonly the orbit (19/45 (42%)) (Table 4, Figure 2).

Four recurring risk factors: malignancy, transplant, diabetes mellitus and illicit drug use, alone or in combination, were present in most (42/45 (93%)) cases. In the few patients (3/45 (7%)) without these risk factors, the invasive infection was due to a skin laceration (n = 1) and pre-existing allergic fungal sinusitis (n = 2) (Table 5).

Malignancy (23/45 (51%)) and diabetes mellitus (18/45 (40%)) were the most common risk factors. Notably, these risk factors often coexisted in patients. For instance, among the 23 patients with malignancy, 10/23 (44%) had received transplants, and 3/23 (13%) also had diabetes mellitus. In the diabetes group, 2/18 (11%) had received transplants, 3/18 (17%) had malignancies and 2/18 (11%) were illicit drug users. Among the five patients with illicit drug use, two-fifths (40%) had diabetes and three-fifths (60%) did not have any other risk factors. A detailed breakdown of these risk factors, either alone or in combination, is provided in the last three columns of Table 5.

The overall mortality in the cohort was 53% (24/45) (Table 6). Of the 24 deaths, 75% (18/24) were secondary to the fungal infection, and 25% (6/24) were due to the underlying disease.

As previously mentioned, the most prevalent risk factors were malignancy and diabetes mellitus. Mortality rates among these groups, when not associated with other risk factors, were 75% (9/12) for malignancy and 27% (3/11) for diabetes. The most common concurrent risk factors were malignancy and transplantation (10/45 (22%)); of these 10 patients, 2/10 (20%) had diabetes and 8/10 (80%) did not. In patients with concurrent malignancy and transplantation, mortality was the highest at 88%. There were no deaths in the group exposed to illicit drug only (three-fifths), but one of two (one-half) patients with concurrent illicit drug use and diabetes died of disease. Illicit drugs included two marijuana users and three patients with the concurrent use of methamphetamines and opioids.

An evaluation for statistically significant correlations between the identified risk factors and the outcome of death was performed using both Pearson’s Chi-squared test and Fisher’s exact tests since the sample was of intermediate size. Odds ratios were calculated to determine the strength of association between the identified risk factors and the outcome of death. The results of these tests are summarized in Table 7. Highly significant correlations between malignancy (*p* < 0.001) and transplant (*p* < 0.009) and death were found, with odds ratios (ORs) of sixteen for malignancy and an OR = 8 for transplant. There was no statistical correlation between diabetes mellitus and illicit drug use and mortality.

The therapies received are summarized in Table 8. Most patients (40/45 (89%)) had at least one surgery including resections of sinonasal and facial tissues (maxillectomies, orbitotomies, or exenterations) and endoscopic debridement. A third (15/45 (33%)) of the patients had multiple/repeated surgical procedures. In 5/45 (11%) patients with dismal prognosis due to the extent of disease, only biopsies not followed by surgery were performed before transitioning to palliative care. In three of these five patients, antifungals were not started; all the remaining patients (42/45 (93%)) received antifungal agents in combination or sequentially.

The most used antifungals were triazoles (35/45 (78%)), frequently preceded or used in combination with liposomal amphotericin B (27/45 (60%)). Among the triazoles, voriconazole was the most used (14/35 (40%)), followed by posaconazole and isavuconazonium (9/35 (26%) each). Micafungin (3/35 (7%)) and topical terbinafine (2/35 (4%)) were added only in a few patients.

**Figure 3:**
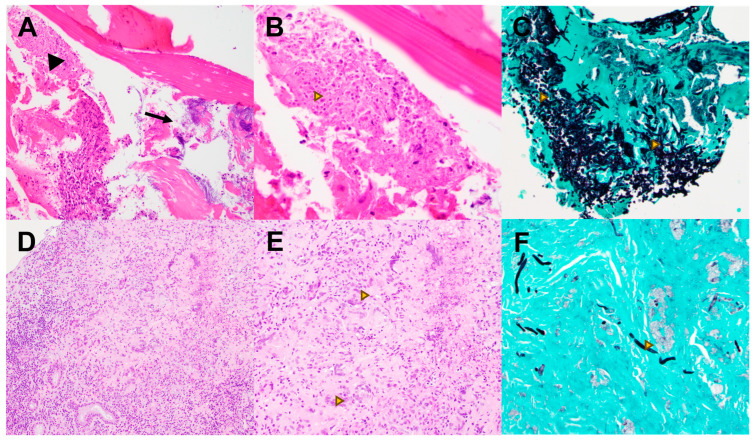
(**A**–**C**): Candidiasis. (**A**) H&E, lower magnification shows *Candida* (arrowhead) and *Actinomyces* (arrow) coinfection complicating a case of osteoradionecrosis of the mandible. (**B**) H&E, higher magnification and (**C**) GMS demonstrate characteristic “spaghetti and meatballs” yeasts and fungal hyphae (arrowheads) much more evident with GMS staining. (**D**–**F**): *Curvularia.* (**D**) H&E, lower and (**E**) higher magnifications show poorly formed granulomas with numerous multinucleated giant cells (arrowhead) involving sinonasal mucosa. (**F**) GMS stain highlights septate hyphae not seen on H&E. A PCR assay confirmed *Curvularia* species.

There were only eight cases of invasive fungal infection involving the mandibular bone. In all these cases, the causative organism was *Candida* (Figure 3). In seven out of eight (88%) cases, the infection was a complication of osteoradionecrosis and was associated with actinomycosis. In a single case (one–eighth (12%)), the infection was associated with a dental implant.

**Figure 4:**
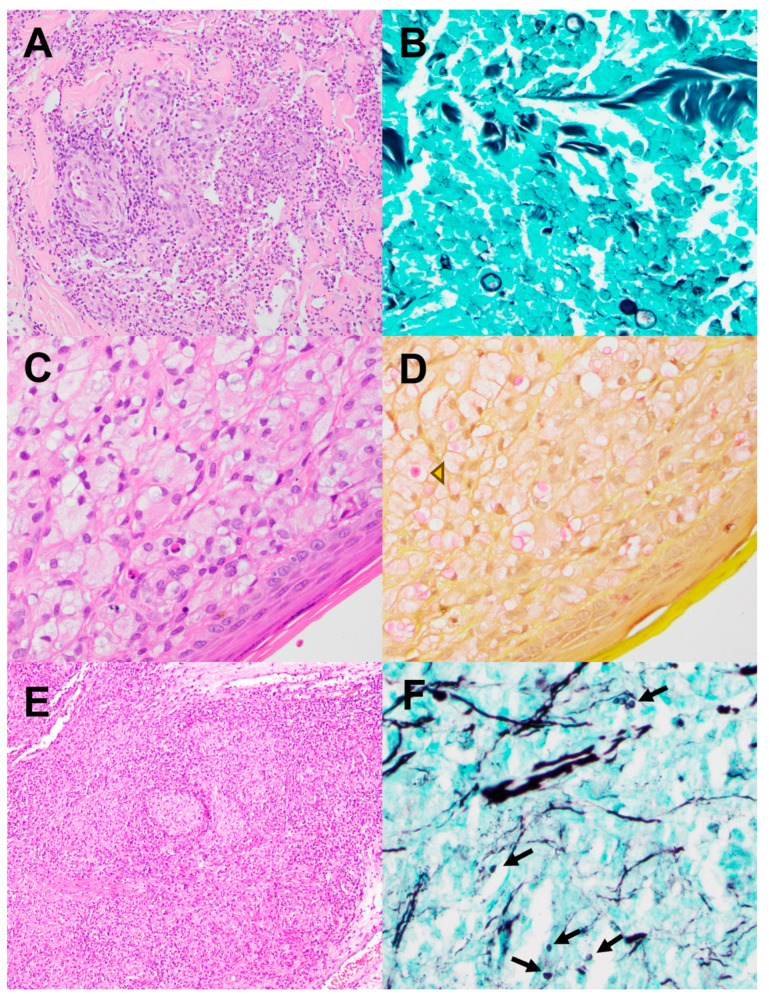
(**A**,**B**): Blastomycosis. (**A**) H&E, lower magnification shows abundant mixed inflammatory cells in postauricular skin. (**B**) GMS stain reveals large budding yeasts (arrowhead). (**C**,**D**): Cryptococcosis. (**C**) H&E slide demonstrates numerous foamy histiocytes and rare eosinophils in the skin of ear. (**D**) Mucicarmine stain highlights the cryptococcal capsule (magenta, arrowhead). (**E**,**F**): Histoplasmosis. (**E**) Extensive non-necrotizing granulomatous inflammation involving the epiglottis. GMS highlights scattered diminutive teardrop-shaped *Histoplasma* yeasts (arrows).

There were only two cases of blastomycosis, a cutaneous infection of the nose and an infection of the external ear and post-auricular skin. In both cases, a penetrating skin injury preceded the infection. A single case of invasive cutaneous cryptococcosis affecting the ear occurred in a 32-year-old male with active HIV/AIDS infection. A single case of histoplasmosis was identified. It affected the larynx of a 73 y.o. man who was an active hunter and was receiving a tumor necrosis factor inhibitor for Crohn’s disease (Figure 4).

## 4. Discussion

We have compiled our 19-year experience with fungal infections of the H&N at a large tertiary university hospital system. At skin and mucosal sites in the H&N, our study expectedly showed that non-invasive cases far outnumbered invasive infections. However, the ratio of non-invasive to invasive cases varied markedly by site: in the oral cavity and oropharynx/esophagus, the ratio was high >29:1, while at the other sites such as the larynx (5:1) and nasal/paranasal cavities (4.5:1) and in the ear/mastoid (2:1), it was low. This variation aligns with the frequency of commensal, asymptomatic and symptomatic infections at these different sites and underscores the inherent efficacy of barrier function across different epithelia, with skin being more protective than squamous mucosa and respiratory mucosa.

Sinonasal infections accounted for most of the invasive infections of the H&N by a ratio of ≥15:1 compared to the other sites, explaining why most of the existing literature refers to sinonasal infections. In these cases, an extension of the infection beyond the sinonasal cavities was present in 73% of the cases and accounted for most of the orbital and base-of-skull infections. These results are similar to those reported in systematic reviews of this subject [5,6]; however, they are likely influenced by referral bias since serious fungal infections require treatment at tertiary centers with large H&N surgical teams able to handle these complex cases [6]. In our cohort, one-third of the patients required multiple procedures, including neurosurgical interventions. The most common organisms were Mucorales and hyaline molds; among these groups, *Mucor* spp. and *Aspergillus* spp. were the most common organisms. Although a specific identification of the organism by culture was attempted in all cases, in 40% of the cases with negative cultures, morphologic examination alone was enough for guiding therapy and specifically requested by infectious disease specialists if an identification had been deferred to culture in the original pathology report. This indicates that pathologists should always provide a presumptive morphologic identification that can be fine-tuned after the results of cultures or PCR become available.

Although fungi are less common causes of infection of the ocular tissues than bacteria and viruses, they are worldwide pathogens involved in infections of the eye, eye adnexa and the orbit [7,8]. The fungi causing oculo-orbital and H&N infections are usually from the *Mucor* and *Aspergillus* genera, which are ubiquitous in soil and vegetable matter around the world, however, characteristic geographical distributions exist [9,10].

Fungal endophthalmitis in immunocompetent individuals usually follows accidental penetrating trauma, especially wood, and less commonly, it is due to the extension of infection from the cornea, eyelid margin, conjunctiva or lacrimal system. Ophthalmic surgeries can lead to contamination with eyelid or conjunctival sac flora or contaminated irrigating solutions. Keratoplasty with contaminated donor storage media may also cause invasive fungal infections [11,12].

Endogenous fungal endophthalmitis is an infrequent but serious complication of systemic mycoses. The most frequent culprit is *Candida*, affecting mainly individuals with systemic debilitating diseases, such as the acquired immunodeficiency syndrome (AIDS), chemotherapy, immunosuppressive therapy, intravenous catheterization and illicit intravenous drug abuse [7,8,11].

The main orbital fungal infections, zygomycosis (most commonly Mucorales) and aspergillosis, are typically extensions from the paranasal sinuses as demostrated in our study. Diabetic ketoacidosis predisposes one to zygomycosis, whereas allergic fungal sinusitis precedes orbital aspergillosis. The principal predisposing factor in mycotic infections of the lacrimal drainage apparatus is a partial or the complete blockage of that system.

The airborne spores enter the body through the respiratory tract. In the nose and paranasal sinuses, the fungus may proliferate, penetrate blood vessels and spread by vascular and direct extension to the orbit [13]. Vascular invasion leads to thrombosing vasculitis and an infarction of the surrounding tissues. Thrombosis of the ophthalmic and/or central retinal artery may result in the extensive infarction of intraocular structures. Orbital cellulitis, with the involvement of the optic nerve and the nerves that pass through the sphenoidal fissure (orbital apex syndrome, Figure 2D), is usually associated with fatal cerebral involvement.

Several *Zygomycetes,* but most commonly *Rhizopus*, cause the clinical syndrome of cerebrorhinoorbital phycomycosis, a highly lethal disease. In adults, 50% of affected patients have poorly controlled diabetes; however, the infection can occur in patients with mild or unrecognized diabetes and occasionally in healthy individuals [9,14]. Additional recognized risk factors, beyond those mentioned before, include alcoholic cirrhosis, ulcerative colitis, extensive burns and deferoxamine therapy.

In tropical and subtropical areas, fungi from the Entomophthorales species may cause cerebrorhinoorbital phycomycosis, most frequently due to the extension of infections originating in the sinonasal passages, leading to characteristic dark, gangrenous lesions in the periorbital skin, nasal mucosa or palate [15].

In our cohort, 93% (42/45) of the patients had one or more of four risk factors: malignancy, transplant, diabetes mellitus and illicit drug use. The first three have an established association with an impaired immune system [16,17], and the last one has an association with increased exposure to contaminated substances [18,19]. In the three cases without these risk factors, a skin laceration was identified as the port of entry of the infection, and in the two remaining cases, the infections originated from fungal sinusitis. Fungal infection-associated mortality had a highly significant association with malignancy and transplant but not with diabetes and illicit drug use. In our cohort, the odds ratio of dying of an infection for malignancy and transplant were 16 and 8, respectively. Fatal infections are commonly associated with severe immunosuppression [17]; in our cohort, 83% of patients had hematologic malignancies, and 17% had somatic malignancies. Among those with a history of transplant, 69% underwent bone marrow transplantation, while the remaining 31% received solid organ transplants [18,19]. Notably, patients with hematologic diseases undergoing bone marrow transplants are particularly susceptible to fatal infections due to the profound immunosuppression affecting both cellular and humoral immune responses because of the disease and/or its treatment. Severe neutropenia is the primary risk factor for *Aspergillus* infections [17], while *Mucor* infections are associated both with neutropenia and diabetes mellitus [16]. These two organisms accounted for most of the infections in our study and reported infections worldwide [5,6,7,9,10,11,12,14]. The overall mortality rate in our cohort was 53%, consistent with rates reported in literature reviews [5,6].

While most invasive infections of the H&N represented an extension of sinonasal disease into the orbit, facial soft tissues and base of the skull, the involvement of the mandible, which comprised 10% of invasive infections, was primarily due to an extension of oral candidiasis into areas of osteoradionecrosis and was frequently associated with *Actinomyces*. This association had also been reported in a previous study [20].

Invasive infections affecting the ear, oral cavity and larynx were exceedingly rare in our study. These infections were associated with specific risk factors, including penetrating injuries, hematologic malignancies, immunosuppressive medications and HIV/AIDS. A large series of laryngeal fungal infections in Vietnam (n = 48) revealed that *Aspergillus* was the most common causative organism, followed by *Candida* [21]. Notably, all patients in this series were immunocompetent, 44% were smokers and the infections were attributed to environmental exposure.

The literature on invasive fungal infections of the oral cavity and the ear is limited and primarily based on literature reviews. In both locations, *Aspergillus* and *Candida* are consistently cited as the most common organisms, particularly affecting immunocompromised individuals [22,23,24]. In our case series, 97% (86 out of 89) of oral cavity fungal infections were non-invasive and caused by *Candida*. All three patients with invasive infections were immunocompromised: two with hematopoietic malignancy and one with somatic malignancy.

Among the patients diagnosed with ear/mastoid infection, three out of ten presented with invasive disease. One of these patients was HIV-positive, while the other was immunocompetent and had a history of environmental exposure. The medical history of the third patient was unavailable in our electronic medical system.

Invasive mycoses in the sinonasal tract are managed with aggressive surgical debridement and systemic antifungals. Pre-surgical cranial and sinus computerized tomography and magnetic resonance imaging are strongly recommended to assess the disease extent and guide treatment plan [24]. Resection should be repeated as needed. Skiada, A, et al. showed that surgical treatment decreased the risk of death caused by zygomycosis by 79% [25]. Liposomal amphotericin B combined with surgery provided the best chance of recovery in this study. In our cohort, 89% of patients had surgical intervention and about one-third had more than one procedure. Triazoles were the most used antifungals preceded or concurrently administered with amphotericin B. Follow-up imaging at 6–12 week intervals after surgery is recommended to evaluate patients’ response and determine the duration of therapy [26]. Post-surgical antifungal therapy is usually prolonged and should be continued until signs and symptoms have resolved and radiologic improvement is evident. For invasive aspergillosis, the average treatment duration is 6–12 weeks, and for mucormycosis, it is usually longer than 6 months [26]. Addressing underlying risk factors should be attempted if feasible, for example, by controlling glucose levels in diabetic patients, minimizing immunosuppression in transplant patients and administering granulocyte infusions to neutropenic patients.

## 5. Conclusions

We have compiled our 19-year experience with invasive fungal infections of the H&N at a large tertiary academic health system. Invasive sinonasal infections were 15 times more common than any other site. Orbital infections, the second most common site, represented an extension of sinonasal infections. These infections were mostly caused by *Aspergillus* and *Mucor* and affected patients with one or several of the following risk factors: malignancy, transplant, diabetes mellitus and illicit drug use. Mortality in our cohort was primarily associated with malignancy and transplant. The third most common site was the mandible. At this site, infections occurred as a complication of osteoradionecrosis and were caused by the coinfection of *Candida* and *Actinomyces*. At other H&N sites, infections were exceedingly rare and associated with clearly identifiable risk factors: penetrating injuries, environmental exposures and immunosuppression. The treatment of invasive fungal infections requires the combination of surgical debridement and antifungals, usually for prolonged periods. Despite recent advances in antifungal therapies, mortality remains high (>50%) for immunocompromised patients with advanced infections.

## Figures and Tables

**Table 1:** Fungal infections of the H&N in the IUSM pathology database for the period 2005–2024.

	Invasive (Inv)		Non-Invasive (Non-Inv)		Ratio Non-Inv/Inv
Site	n	%	n	%	
Sinonasal	45	58.4	203	60.4	4.5
Ear/mastoid	3	3.9	7	2.1	2.3
Larynx	2	2.6	10	3.0	5
Oral cavity	3	3.9	86	25.6	28.3
Oropharynx/esophagus	0	0	30	8.9	>30
Base of skull	2	2.6	N/A		N/A
Orbit	14	18.2	N/A		N/A
Mandible	8	10.4	N/A		N/A

N/A: not applicable. Non-invasive infections do not occur at these sites.

**Table 2:** Invasive sinonasal infections, demographics.

Demographics (N = 45)	
Age (yrs.)	n (%)
≤18	3 (7)
19–≤40	9 (20)
>40	33 (73)
Gender	n (%)
M	31 (69)
F	14 (31)

**Table 3:** Fungus identification in invasive sinonasal infections.

Morphology	Genus/Species	n (%)	N (%)
Mucorales	*Mucor* sp. *Rhizopus* sp. *Rhizomucor* sp. *Sincephalastrum racemosum*	18 (64) 5 (18) 4 (14) 1 (4)	28 (51)
Hyaline molds	*Aspergillus* spp. *Fusarium* sp. *Scedosporium* sp.	14 (88) 1 (6) 1 (6)	16 (29)
*Candida*	*C. albicans* *C. parasilopsis* *C. glabrata* *C. dubliniensis* *Candida* sp.	2 (33) 1 (17) 1 (17) 1 (17) 1 (17)	6 (11)
Dematiaceous	*Curvularia* sp. *Dematiaceous* sp.	1 (50) 1 (50)	2 (4)
Other	*Blastomyces dermatitides* *Kluyveromyces marxianus* Mycelia sterilia	1 (33) 1 (33) 1 (33)	3 (6)

**Table 4:** Extent of invasive sinonasal infections.

Extension	n	%
Sinonasal only	12	27
Beyond sinonasal	33	73
Extrasinonasal extension		
Orbit	19	42
Intracranial	5	11
Base of skull	5	11
Facial soft tissue	3	7
Palate	3	7
Nasopharynx	2	4
Oropharynx	1	2

**Table 5:** Frequency of risk factors and death outcome in invasive sinonasal infections.

Risk Factor	Subgroup n (%)		n (%)	Dead n (%)	All Risk Factors	n (%)	Dead n (%)
Malignancy (Malig.)	Hematolymphoid	19 (83)	23 (51)	19 (83)	Malig. only Malig. + TX Malig. + DM Malig. + drugs Malig. + TX + DM TX only TX + DM TX + drugs DM only DM + drugs Drugs only	12 (27) 8 (18) 1 (2) 0 2 (4) 1 (2) 2 (4) 0 11 (24) 2 (4) 3 (7)	9 (75) 7 (87.5) 1 (100) 0 2 (100) 1 (100) 1 (50) 0 3 (27.3) 1 (50) 0
	Somatic	4 (17)					
Transplant (TX)	Bone marrow	9 (69)	13 (29)	11 (85)			
	Pancreas/kidney	1 (8)					
	Pancreas	1 (8)					
	Kidney	1 (8)					
	Liver	1 (8)					
Diabetes mellitus (DM)	Type 1	5 (28)	18 (40)	7 (39)			
	Type 2	13 (72)					
Illicit drug	Marijuana	2 (40)	5 (11)	1 (20)			
	Methamph. *	3 (60)					
	Opioids *	3 (60)					
None	-		3 (7)	0			

* Percentages reflect concurrent use of more than one drug.

**Table 6:** Invasive sinonasal infections, outcomes.

Outcome	n (%)	Cause Specific Mortality	n (%)
Alive Dead	21 (47) 24 (53)	Fungal infection Malignancy	18 (75) 6 (25)

**Table 7:** Sinonasal infections. Significance/strength of association between risk factors and outcome of death.

Risk Factor	Pearson Chi2 Test (Two-Sided)	Fisher’s Exact Test (Two-Sided)	Odds Ratio	95% Confidence Interval
Malignancy	<0.001 ***	<0.001 ***	16.150	3.718–70.142 *
Transplant	0.007 **	0.009 **	8.038	1.523–42.430 *
Diabetes mellitus	0.113	0.138	0.374	0.110–1.278
Illicit drug	0.113	0.169	0.185	0.019–1.805

This is the output from SPSS to stress statistical significance: for *p*-value * <0.05, ** < 0.01, *** < 0.001, for the CI, it denotes statistical significance.

**Table 8:** Treatment for invasive sinonasal infections.

Therapy	n	%	Triazoles	n (%)
Surgery			Voriconazole	14 (40%)
One surgery	25	56	Itraconazole	1 (3%)
Multiple surgeries	15	33	Posaconazole	9 (26%)
No surgery (biopsy only)	5	11	Isavuconazole	9 (26%)
Antifungals			Fluconazole	2 (6%)
At least one antifungal	42	93		
No antifungals	3	7		
Specific antifungals *				
Amphotericin B	27	60		
Triazoles (see right columns)	35	78		
Micafungin	3	7		
Terbinafine	2	4		

* Percentages reflect concurrent or consecutive use of more than one drug.

## Data Availability

The raw data supporting the conclusions of this article will be made available by the authors on request.
